# A core outcomes set for clinical trials of interventions for young adults with type 1 diabetes: an international, multi-perspective Delphi consensus study

**DOI:** 10.1186/s13063-017-2364-y

**Published:** 2017-12-19

**Authors:** Molly Byrne, Anthony O’Connell, Aoife M. Egan, Sean F. Dinneen, Lisa Hynes, Mary Clare O’Hara, Richard I. G. Holt, Ingrid Willaing, Michael Vallis, Christel Hendrieckx, Imelda Coyne

**Affiliations:** 1 Health Behaviour Change Research Group, School of Psychology, National University of Ireland, Galway, Ireland; 2 School of Medicine, National University of Ireland, Galway, Ireland; 3 Endocrinology and Diabetes Centre, Galway University Hospitals, Galway, Ireland; 4 Human Development and Health Academic Unit, University of Southampton Faculty of Medicine, Southampton, UK; 5 Health Promotion Research, Steno Diabetes Center Copenhagen, Copenhagen, Denmark; 6 Department of Family Medicine, Dalhousie University, Halifax, NS Canada; 7 School of Psychology, Deakin University, Geelong, VIC Australia; 8 The Australian Centre for Behavioural Research in Diabetes, Diabetes Victoria, Melbourne, VIC Australia; 9 Trinity College Dublin, School of Nursing and Midwifery, Dublin, Ireland

**Keywords:** Clinical diabetes, Healthcare delivery, Type 1 diabetes, Young adults, Interventions, Core outcome set, Self-management, Randomised controlled trials

## Abstract

**Background:**

Achieving consensus from a range of relevant stakeholders about an agreed set of core outcomes to be measured and reported as a minimum in clinical trials has the potential to enhance evidence synthesis and make findings more relevant and applicable. Intervention research to improve outcomes for young adults with type 1 diabetes (T1DM) is hampered by inconsistent use of outcome measures. This population frequently struggles to manage their condition and reports suboptimal clinical outcomes. Our aim was to conduct an international, e-Delphi consensus study to identify a core outcome set (COS) that key stakeholders (young adults with T1DM, diabetes health professionals, diabetes researchers and diabetes policy makers) consider as essential outcomes for future intervention research.

**Methods:**

Using a list of 87 outcomes generated from a published systematic review, we administered two online surveys to a sample of international key stakeholders. Participants in the first survey (survey 1; *n* = 132) and the second survey (survey 2; *n* = 81) rated the importance of the outcomes. Survey 2 participants received information on total mean rating for each outcome and a reminder of their personal outcome ratings from Survey 1. Survey 2 results were discussed at a consensus meeting and participants (*n* = 12: three young adults with T1DM, four diabetes health professionals, four diabetes researchers and one diabetes policy maker) voted on outcomes. Final core outcomes were included provided that 70% of consensus group participants voted for their inclusion.

**Results:**

Eight core outcomes were agreed for inclusion in the final COS: measures of diabetes-related stress; diabetes-related quality of life; number of severe hypoglycaemic events; self-management behaviour; number of instances of diabetic ketoacidosis (DKA); objectively measured glycated haemoglobin (HbA_1C_); level of clinic engagement; and perceived level of control over diabetes.

**Conclusions:**

This study is the first to identify a COS for inclusion in future intervention trials to improve outcomes for young adults with T1DM. Use of this COS will improve the quality of future research and increase opportunities for evidence synthesis. Future research is necessary to identify the most robust outcome measure instruments.

**Electronic supplementary material:**

The online version of this article (doi:10.1186/s13063-017-2364-y) contains supplementary material, which is available to authorized users.

## Background

Type 1 diabetes (T1DM) requires intensive self-monitoring of blood glucose and self-management, including administering insulin, regulating diet and exercise, to maintain optimal glycaemic control [1]. Young adults, aged 15–30 years, are one group of individuals consistently identified as being at higher risk of disengagement from diabetes self-management and adherence to diabetes medication [2]. Young adults have been identified as being at higher risk than other groups of suboptimal glycaemic control [3] and higher incidence of ketoacidosis (DKA) [4]. Young adulthood is often a time of transition during which diabetes self-management can be particularly difficult, with common lifestyle challenges including moving away from the family home for the first time, beginning employment or university, and transitioning from paediatric to adult healthcare services [5, 6].

Effective interventions to improve self-management and outcomes for young adults with T1DM are needed. Systematic reviews of interventions have suggested that there have been relatively few trials and the quality of this research is often low [5, 7]. Furthermore, evidence synthesis within systematic reviews is hampered by the use of diverse outcomes and measures across intervention studies [7].

Within clinical trials research, there is growing awareness that insufficient attention has been paid to what outcome measures are selected [8]. Frequently, trials do not include outcomes which are considered most important to young adults and professionals making decisions about healthcare, limiting the relevance and applicability of research. In addition, synthesizing evidence from trials to determine the most effective interventions is frequently hampered by heterogeneity in outcome measurement [9].

To address this problem, trialists advocate the identification of core outcome sets (COS), defined as agreed standardised collections of outcomes which should be measured and reported in all trials, for specific clinical areas [10]. Such COS are considered to be a minimum set of outcomes, which should be measured and reported in all trials. The involvement of key stakeholders, including patients, the public and healthcare professionals, in the identification of COS can ensure that outcomes are relevant to all stakeholder groups [9]. Previous COS studies with patients or the public have identified outcomes that were not previously identified by other stakeholders [11]. The COMET (Core Outcome Measures in Effectiveness Trials) Initiative (see: http://comet-initiative.org/) brings together people interested in the development and application of agreed standardised sets of outcomes, which has led to a growing number of published COS studies [12–18]. No COS has yet been produced for clinical trials of interventions for young adults with T1DM.

The aim of this study was to identify a COS for intervention trials aiming to improve clinical, behavioural or psychosocial outcomes for young adults with T1DM. A recent review of such interventions reported a wide range of different outcomes being used across trials and recommended the development of a COS for future research [7]. We have conducted and reported our COS study in accordance with recently published guidelines [8].

## Methods

We conducted an international, multi-perspective Delphi consensus study, which involved three phases: (1) generation of a list of all possible relevant outcomes; (2) an electronic Delphi survey, which contained two rounds; and (3) a consensus meeting to agree a final COS. A Delphi study involves several sequential rounds of data collection and analysis in order to collate the opinions of participants into a group consensus on a particular topic. After each round, responses are analysed and redistributed to participants for further comment in successive rounds [12]. Ethical approval was granted from the University Research Ethics Committee on 29 January 2016 (ref: CA1427).

### Participants

Participants were recruited using multiple routes. Young adults with T1DM (aged 15–30 years), diabetes health professionals, diabetes researchers and policy makers in diabetes services were included to ensure that our COS was comprehensive and clinically relevant. The international research team facilitated the recruitment process, by distributing the study invite via email to lists of diabetes researchers, health professionals and policy networks to which they had access. Young adults with T1DM were invited via support groups, diabetes services or through other methods accessible to members of the study team. For example, in Ireland, the study invite was circulated to Diabetes Ireland, a national charity dedicated to supporting and educating people with diabetes. The study was more widely announced via social media channels, for example, invitations were posted on Facebook accounts of online support groups for people with T1DM. Snowball sampling was used and participants were invited to convey the study details to other individuals who may have relevant expertise to participate in the study.

The invitation email included information about the study aims, methods and research team, and a link to the survey. Informed consent was obtained from each participant upon online registration for the survey, by providing participant information and requesting that participants indicate consent by clicking on the consent box. The importance of completing both rounds of the survey was emphasised and generic reminder emails were distributed to round 1 participants to increase completion of round 2. A unique identifier was assigned to each participant tracked to their email address which allowed linkage of participants in rounds 1 and 2. The study information text used to introduce Surveys 1 and 2 can be seen in the Additional file 1.

### Phase 1: Generation of a list of all possible relevant outcomes

An initial list of possible relevant outcomes was generated from a previously conducted systematic review of interventions to improve clinical, behavioural or psychosocial outcomes for young adults (aged 15–30 years) with T1DM [7]. This list was reviewed and discussed by the research team and outcomes were collapsed where possible to create a list of distinct outcomes. The team also had the opportunity to suggest any additional outcome measures they thought were potentially important but not included in the list. A final list of 87 outcomes was agreed upon.

### Phase 2: Electronic Delphi survey

The Delphi surveys were created using SurveyMethods online survey software (https://www.surveymethods.com/). The surveys were piloted to ensure clarity and understanding with three young adults (who were diabetes service users) and one researcher. Within both surveys, participants were shown the list of 87 outcomes, grouped into seven domains for ease of survey completion: Lifestyle; Quality of life; Diabetes clinics; Medical; Blood glucose; Treatment preferences in relation to diabetes; and Intervention-related outcomes.

In survey 1, participants were asked to rate how important they considered each of the outcomes, on a scale of 1–9, where 9 was the most important. Survey 1 included a brief demographic questionnaire, in which respondents provided information on: country of residence; gender; whether they had been diagnosed with T1DM; and the stakeholder group to which they belonged (young adults with T1DM, diabetes health professionals, diabetes researchers or people who inform policy on diabetes services). As roles are not mutually exclusive, participants could indicate that they belonged to more than one group. Survey 1 was live for one month (from 29 April 2016 to 29 May 2016).

Four weeks after they completed Survey 1, all participants were followed up by email and invited to complete Survey 2. In Survey 2, participants received information about the average rating for each outcome by all participants in Survey 1 and a reminder of their own Survey 1 rating. Participants were asked to re-rate the importance of each outcome with knowledge of their individual as well as the groups’ previous ratings. The same Likert-type rating scale in the range of 1–9 was used. Survey 2 closed on 17 June 2016.

### Phase 3: Consensus meeting

A 3-h consensus meeting was held in Galway, Ireland, in June 2016 with the aim to agree on a final COS, informed by ratings from the Delphi survey. Participants for this meeting were sampled purposively to get a balanced representation for each of the stakeholder groups and a reasonable geographical spread. The sample was drawn from participants who had completed both survey rounds. Survey 2 data were used to generate discussion and move towards consensus. Before the meeting, using Survey 2 data, each outcome was categorised as category A (high agreement and high support), category B (low agreement and mixed support) or category C (high agreement and low support). Category C outcomes were excluded from further discussion and were not considered for inclusion in the final COS. See Table 1 for further detail of categorisation criteria.

**Table 1:** Criteria for categorising outcomes based on Delphi Survey 2 data for the consensus meeting

Category	Criteria required
Category A: High agreement and high support	Rated by ≥ 70% of participants as ≥ 8
Category B: Low agreement and mixed support	Rated by < 70% of participants as ≥ 8 and rated by < 70% of participants scored as ≤ 6
Category C: High agreement and low support	Rated by ≥ 70% of participants as ≤ 6

The meeting was structured into three parts. First, review and discussion of outcomes for which there had been low agreement in survey 2 (category B outcomes). These outcomes were discussed by domain and participants were asked to share their views with the group about why they considered the outcome should, or should not, be considered for inclusion in the final decision-making discussion and voting. Second, re-rating of category B outcomes on a paper survey, using the same Likert-type rating scale in the range of 1–9. These data were entered into a Microsoft Excel software database at a computer within the room. Data were entered by one person, and checked by a second. Those outcomes which at least 70% of consensus group participants had rated as 8 or higher in the re-rating process, were named ‘new category A outcomes’ and were retained for inclusion in the final decision-making discussion and voting phase. The final phase involved the discussion and voting of category A outcomes for inclusion in the final COS.

During this phase, all original category A outcomes (from Survey 2) and new category A outcomes (from the consensus meeting re-rating) were written onto large sticky notes and posted on a wall in the room. Each outcome was discussed in turn, followed by a vote (by hand-raising) as to whether participants believed the outcome should be included in the final COS. A member of the research team recorded the number of votes each outcome received on the sticky note.

Those outcomes voted for inclusion by at least 70% of consensus group participants (≥9) were grouped together and presented on a separate part of the wall under the heading ‘final core outcome set’. After all outcomes had been voted on, and the total COS was reviewed, the group were given another opportunity to comment on included items and indicate if they believed the COS was comprehensive. Following this discussion, any outcomes which were discussed at this phase were voted on and the same inclusion criteria were applied (i.e. outcomes which at least 70% of consensus group participants voted for were retained in the final COS). Any category A outcomes not included in the final COS, became part of a list categorised as ‘supplementary outcomes’.

## Results

**Fig. 1:**
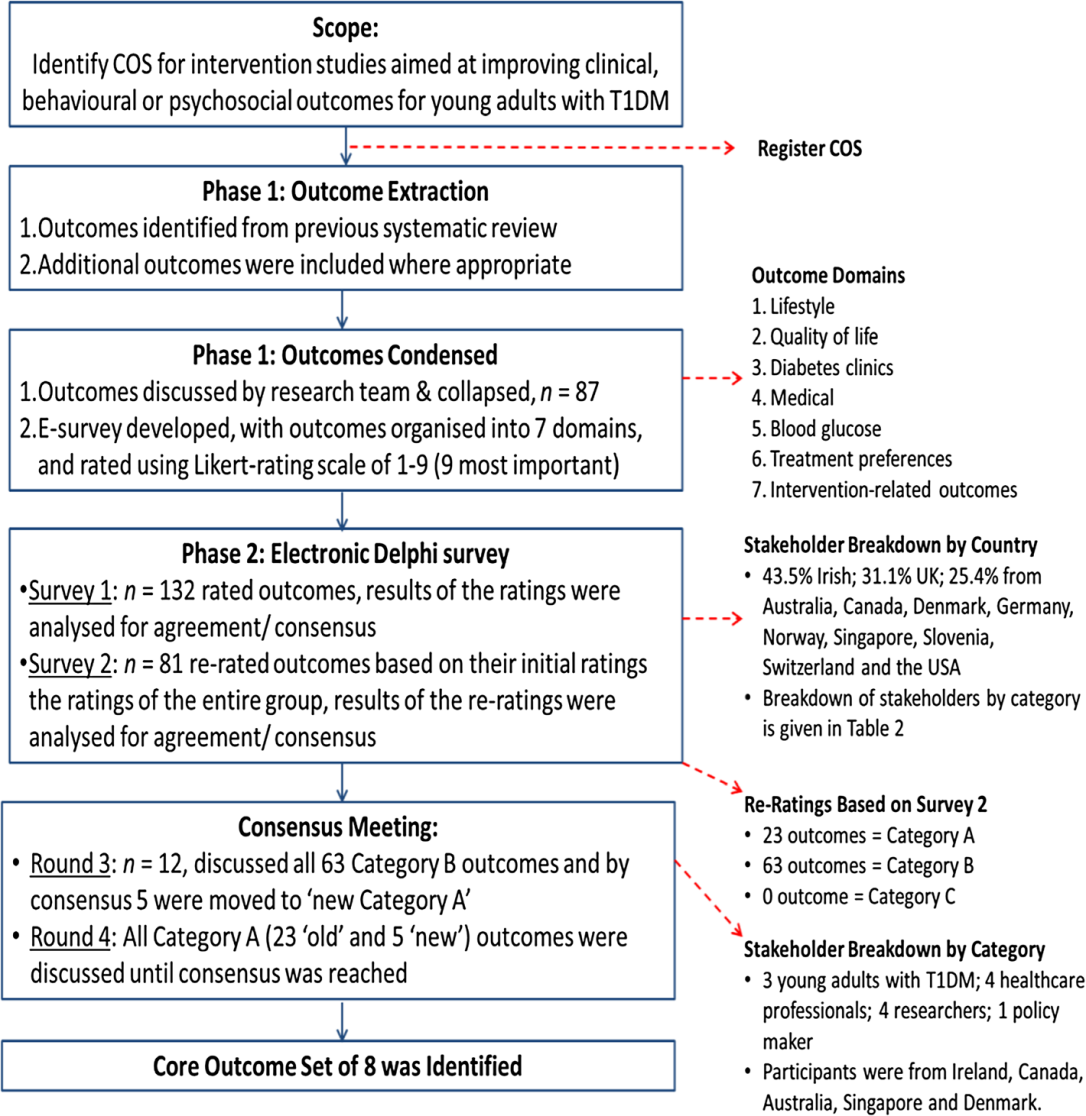
*Flowchart* of the study methodology

Figure 1 shows a flowchart of the study methodology and summarises outcomes at each phase. Details of Delphi survey participants (stakeholder group and gender) at rounds 1 (*n* = 132) and 2 (*n* = 81), as well as consensus meeting participants (*n* = 12), can be seen in Table 2. Of the 12 people who participated in the consensus meeting, eight were women and four were men. The group included three young adults with T1DM, four diabetes health professionals, four diabetes researchers and one diabetes policy maker. Participants were from Ireland (n = 8), Canada (*n* = 1), Australia (*n* = 1), Singapore (*n* = 1) and Denmark (*n* = 1).

**Table 2:** Details of participants in Delphi survey rounds 1 and 2 and consensus meeting (stakeholder group, gender and country of residence)

	Round 1 n (% of total) (*n* = 132)	Round 2 n (% of total) (*n* = 81)	Consensus meeting n (*n* = 12)
*Stakeholder group*			
Young adults with T1DM	34 (25.8)	17 (21.0)	3
Diabetes researchers	30 (22.7)	24 (29.6)	4
Diabetes health professionals	76 (57.6)	49 (60.5)	4
Diabetes policy makers	2 (1.5)	1 (1.2)	1
*Gender*			
Male	28 (21.2)	22 (27.2)	4
Female	104 (78.8)	59 (72.8)	8
*Country of residence*			
Australia	1 (0.8)	1 (1.2)	1
Canada	6 (4.5)	6 (7.4)	1
Denmark	7 (5.3)	5 (6.2)	1
England	11 (14.5)	3 (3.7)	-
Germany	2 (1.5)	1 (1.2)	-
Ireland	64 (48.5)	40 (49.4)	8
N. Ireland	9 (6.8)	4 (4.9)	-
Norway	1 (0.8)	-	-
Scotland	6 (4.5)	-	-
Singapore	1 (0.8)	1 (1.2)	1
Slovenia	1 (0.8)	-	-
Switzerland	1 (0.8)	1 (1.2)	-
UK	19 (14.4)	18 (22.2)	-
USA	2 (1.5)	1 (1.2)	-
Wales	1 (0.8)	-	-

Outcome ratings at rounds 1 and 2 are summarised in Additional file 2: Tables S1–S7. Based on the categorisation of Survey 2 data, the 87 outcomes were categorized as follows: 23 category A outcomes; 63 category B outcomes; and 0 category C outcomes (see Table 3).

**Table 3:** Categorisation of outcomes by domain as A, B or C,^a^ based on Delphi Survey 2 ratings

Domain	Outcomes in category A (n)	Outcomes in category B (n)	Outcomes in category C (n)
Lifestyle	0	8	0
Quality of life	7	5	0
Diabetes clinics	0	10	0
Medical	5	6	0
Blood sugar	3	11	0
Treatment preferences in relation to diabetes	2	15	0
Intervention-related	6	8	0
Total	23	63	0

^a^Category A: strong agreement and strong support; category B: low agreement and mixed support; category C: high agreement and low support. For further details on category definitions, see Table 2

Based on the discussion and re-rating of category B outcomes during the consensus meeting, five category B outcomes were re-categorised as ‘new category A outcomes’. Three of these were deemed new category A outcomes as they had reached the consensus criteria on re-rating. These were: ‘Number of missed clinic appointments’ (nine participants rated as 8+); ‘Number of events of severe hypoglycaemia’ (11 participants rated as 8+); and ‘Perceived level of control over diabetes’ (eight participants rated as 8+). Two further items were included as meeting participants argued strongly for their inclusion. These were: ‘Body mass index (BMI)’; and ‘Perceived levels of barriers to treatment’. The group agreed to allow these two additional variables to be included in the ‘new category A outcomes’ list. The re-rating data for these variables can be seen in Table 4.

**Table 4:** Re-rating data for ‘new category A’ outcomes^a^ (i.e. outcomes which were later included in the voting for inclusion phase) during the consensus meeting

Outcome name	Number rating outcome measure 8 or higher (*n* = 12)	Mean rating	Standard deviation
Body mass index (BMI)^b^	3	6.33	2.06
Missed clinic appointments (n)	9	7.75	1.36
Events of severe hypoglycaemia (n)	11	8.67	0.65
Perceived levels of barriers to treatment^b^	7	6.83	2.04
Perceived level of control over diabetes^c^	8	7.50	2.28

^a^New category A outcomes are those outcomes categorised as outcome B based on Delphi Survey 2 ratings, but subsequently received sufficient support through discussion and re-rating at the consensus meeting to warrant their inclusion in the set of outcomes voted on for inclusion in the final COS

^b^While the outcome ‘BMI’ was well below the cut-off for inclusion as a new category A outcome, one participant argued passionately for its inclusion and subsequently consensus group participants agreed for its inclusion in the voting phase

^c^While this variable was one vote below the cut-off for inclusion as a new category A outcome, a number of participants argued for its inclusion in the voting phase and consensus group participants agreed

The final phase of the consensus meeting, which was the decision-making discussion and voting phase, lasted for approximately 1 h. During this phase, 23 ‘original category A outcomes’ and five ‘new category A outcomes’ were discussed and voted upon. Additional file 2: Table S8 shows levels of support for each of the ‘supplementary important outcomes’, i.e. the category A outcomes that were not voted for inclusion in the COS. The final list was agreed through discussion: the names of some outcomes were changed slightly to clarify the meaning, some outcomes were collapsed together and assumed under one outcome title and other outcomes were added. Outcomes whose name was changed to clarify meaning included: ‘Measure of diabetes related burden’, which became ‘Measure of diabetes related burden and stress’; ‘Glycated haemoglobin (HbA_1c_) measured by a researcher’ became ‘Objectively measured HbA_1c_’; ‘Number of missed clinic appointments’ became ‘Level of clinic engagement’; and ‘Number of hospitalisations and readmissions for diabetic ketoacidosis (DKA)’ was renamed as ‘Number of instances of diabetic ketoacidosis (DKA)’. Two outcomes—‘Number of events of severe hypoglycaemia’ and ‘Severity of events of hypoglycaemia’—were discussed and participants agreed that these could be subsumed under the one outcome heading ‘Number of events of severe hypoglycaemia’. The outcome ‘Quality of life’ was discussed and participants agreed that ‘Diabetes-related quality of life’ was more suitable as a core outcome, so this outcome was included as an additional outcome and voted upon.

When the final COS was presented and reviewed, there was a strongly expressed view that the COS was lacking some aspect of self-management behaviour. Participants agreed to add this outcome (‘Self-management behaviour’) and it was voted to be included in the final COS.

Table 5 shows levels of support for each outcome in the final COS, containing eight outcomes: measures of diabetes-related burden or stress; diabetes-related quality of life; number of severe hypoglycaemic events; self-management behaviour; number of instances of diabetic ketoacdidosis (DKA); objectively-measured glycated haemoglobin (HbA_1C_); level of clinic engagement; and perceived level of control over diabetes.

**Table 5:** Final COS with level of support in the final voting phase

Outcome	n voting for inclusion of outcome in the final COS (*n* = 12)
1. Measures of diabetes-related burden or stress	12
2. Diabetes-related quality of life	12
3. Number of events of severe hypoglycaemia	11
4. Self-management behaviour	11
5. Number of instances of diabetic ketoacdidosis (DKA)	10
6. Objectively measured glycated haemoglobin (HbA1c)	10
7. Level of clinic engagement	9
8. Perceived level of control over diabetes	9

## Discussion

This is the first attempt to produce a COS for intervention trials aiming to improve clinical, behavioural or psychosocial outcomes for young adults (aged 15–30 years) with T1DM. We sought the views of an international sample of young adults with T1DM, diabetes health professionals, diabetes researchers and people who inform policy on diabetes in a Delphi consensus process and agreed a final COS containing eight outcomes: measures of diabetes-related burden or stress; diabetes-related quality of life; number of severe hypoglycaemic events; self-management behaviour; number of instances of diabetic ketoacdidosis (DKA); objectively-measured glycated haemoglobin (HbA_1C_); level of clinic engagement and perceived level of control over diabetes. We suggest that future research evaluating interventions in T1DM should assess and report these outcomes. As with all COS, these outcomes are proposed to be included as a minimum, but additional outcomes may be included as appropriate to different interventions and settings.

Our COS covers a broad range of aspects of diabetes, including stress, quality of life, medical or biological markers, self-management behaviour, level of engagement with health services and diabetes management. The fact that stress and quality of life were ranked during the consensus process as more important than measures of metabolic control (such as glycated haemoglobin), supports previous research which has emphasised the need to broaden the types of measures which are traditionally used [19, 20]. Selecting outcomes based on the consensus methods we have used provides a more evidence-based approach to outcome selection than methods generally used, such as simply measuring what has been measured in previous research [21]. Future research is now needed to identify the most appropriate outcome measures for each of these outcomes. Recent guidance has been published offering guidance on this process [22].

These strengths notwithstanding, the present study is not without its limitations. Although Delphi processes have been recommended as an ideal approach to identify which outcomes to measure in clinical trials [21], they have also been criticised regarding the ambiguities which exist around the issue of defining consensus and expertise [23]. While the study aimed to have a reasonably balanced number of participants from all stakeholder groups, this did not prove possible. Policy makers and young adults with T1DM were difficult to recruit and were under-represented in the study. Furthermore, it is possible that the young adult participants were not representative of all young adults, as they are likely to be more active in acquiring information about T1DM and therefore more engaged. Although we attempted to achieve an international sample in our study, the sample came largely from English-speaking countries, predominantly Ireland. We must use caution in claiming the international generalisability of these findings, especially as non-English-speaking and developing countries are under-represented; future research would be useful to test the international relevance of the outcomes identified as core within our study. Another limitation of the study is that by utilising mailing lists and social media to recruit participants it was impossible to calculate the recruitment rate for the study. The reasonably high level of attrition between rounds 1 and 2 of the study also limits the validity of the study. The final consensus meeting was only open to a small number of participants, again limiting generalisability of the findings. In addition, social and peer pressures may have influenced voting behaviour of consensus group participants, as voting was public involving raising hands to indicate support for outcomes.

## Conclusion

In conclusion, albeit with limitations, this study was an important attempt to identify a COS for intervention research for young adults with T1DM. It provides guidance about what outcomes are important to young adults with T1DM and other key stakeholders. We sought the views of a sample of young adults with T1DM, diabetes health professionals, diabetes researchers and policy makers in diabetes services. This COS will be useful for future intervention trials in this area, encouraging a more coordinated approach to intervention research in the future than currently exists and facilitating more meaningful synthesis of research findings. It is worth noting that any COS identified is a dynamic, rather than fixed, entity, which will evolve as research is conducted. Future research is needed to replicate the findings from this COS study, in particular testing its international generalisability, and to determine and provide guidance on the best outcome measures to select to measure these variables.

## Additional files

Additional file 1: The study information text used to introduce Surveys 1 and 2. (DOCX 20 kb)

Additional file 2: Table S1. Lifestyle domain outcome ratings from Delphi Surveys 1 and 2. **Table S2.** Quality of life domain outcome ratings from Delphi Surveys 1 and 2. **Table S3.** Diabetes clinic-related domain outcome ratings from Delphi Surveys 1 and 2. **Table S4.** Medical domain outcome ratings from Delphi Surveys 1 and 2. **Table S5.** Blood glucose domain outcome ratings from Delphi Surveys 1 and 2. **Table S6.** Treatment adherence domain outcome ratings from Delphi Surveys 1 and 2. **Table S7**: Intervention-related domain outcome ratings from Delphi Surveys 1 and 2. **Table S8**: Level of support in the final voting phase of the consensus meeting for supplementary important outcomes*. (DOCX 28 kb)

## Abbreviations

COMET: Core Outcome Measures in Effectiveness Trials Initiative
COS: Core outcome set
RCT: Randomised controlled trial
T1DM: Type 1 diabetes mellitus
