# Supplementary material for: A core outcomes set for clinical trials of interventions for young adults with type 1 diabetes: an international, multi-perspective Delphi consensus study
Source: Trials. 2017 Dec 19;18:602. doi: 10.1186/s13063-017-2364-y (PMC5735534; doi:10.1186/s13063-017-2364-y)
Supplement: Supplementary file 1 — The study information text used to introduce Surveys 1 and 2. (DOCX 20 kb) [file 13063_2017_2364_MOESM1_ESM.docx]

**Core Outcome Measures in Type 1 Diabetes Part 1**

**Managing Type 1 Diabetes during young adulthood (18-30) can be hard due to the demands of self-management and the busy lifestyles of young adults. As a result, a lot of research has been carried out with young adults with Type 1 Diabetes which has found a variety of different effects.**

**We are an international group of researchers and clinicians who are interested in improving research with young adults with Type 1 Diabetes. The research team is led by Dr Molly Byrne, a Senior Researcher in the National University of Ireland Galway. The research has been funded by the Irish Research Council.**

**We are interested in learning more about what interventions are most effective to improve outcomes for young adults with Type 1 Diabetes. However, research in this area includes many different outcome measures and this makes it hard to compare studies.**

**The aim of our research, is to get views about what are the most important outcomes to measure in research studies. It is important that we get the views of different types of people: people with Type 1 Diabetes, diabetes health professionals, diabetes researchers and people who inform policy on diabetes services.**

**If you agree to take part in this study, you will be asked to fill in two online surveys: one now, and the second about a month from now. The survey should take you no more than 10 minutes to fill in.**

**All information you provide will be treated confidentially. You will not be identified in any report or publication of the study findings. However, as we need to collect e-mail addresses in order to contact you about filling in the second survey. These will not be shared with anyone beyond the research team.**

**In the first survey, you will be presented with a number of different outcomes (XX in total) that have been used in research. You will be asked to tell us how important you think these outcomes are on a scale from 1 (not at all important) to 9 (very important). We are interested in YOUR views, so there are no right or wrong answers.**

**For the second survey, you will get an e-mail with your scores from the first survey and you will be shown the average scores given by others taking part in the study. In the survey, you will be asked again to rate the importance of the outcomes as before.**

**If you have any questions about the study please contact the researcher, Anthony O’Connell at:** [**anthony.oconnell@nuigalway.ie**](mailto:anthony.oconnell@nuigalway.ie)**.**

**If you understand this information and agree to take part please tick this box. This will bring you into the first survey.**

**On the next page you will see a number of different outcome measures that have been used in past research, sorted into a number of different categories. You will be asked to give your opinion as to how important you think each outcome is on a scale from 1 (not at all important) to 9 (very important).**

**Core Outcome Measures in Type 1 Diabetes Part 2**

### * 1. Thank you for completing the first round of this study looking at core outcome measures for research studies conducted with young adults with Type 1 Diabetes. You are now invited to take part in the second round of the study which involves filling out another survey, which should take between 10 and 15 minutes to fill in. We would appreciate it if you could complete this survey by June 10th.

**All information you provide will be treated confidentially. You will not be identified in any report or publication of the study findings. However, we need to collect e-mail addresses in order to match your answers on this round of the study to your answers in round 1 (This is the e-mail address to which you were sent the link to this survey). These will not be shared with anyone beyond the research team.**

**In this survey, you will once again be presented with a number of different outcomes (77 in total) that have been used in research. You will be asked to tell us how important you think these outcomes are on a scale from 1 (not at all important) to 9 (very important). You will be shown the average score from all participants in the study and you can decide if you want to change your answers after reflecting on these average scores. We are interested in YOUR views, so there are no right or wrong answers.**

**You have been sent an e-mail with your scores from the first survey attached as a pdf so you can also look at these while filling out this survey.**

**If you have any questions about the study please contact the researcher, Anthony O’Connell at:** [**anthony.oconnell@nuigalway.ie.**](mailto:anthony.oconnell@nuigalway.ie)

**If you understand this information and agree to take part please tick this box. This will bring you into the survey.**

m I agree to take part in the study
