# Supplementary material for: A core outcomes set for clinical trials of interventions for young adults with type 1 diabetes: an international, multi-perspective Delphi consensus study
Source: Trials. 2017 Dec 19;18:602. doi: 10.1186/s13063-017-2364-y (PMC5735534; doi:10.1186/s13063-017-2364-y)
Supplement: Supplementary file 2 — Lifestyle domain outcome ratings from Delphi Surveys 1 and 2. Table S2. Quality of life domain outcome ratings from Delphi Surveys 1 and 2. Table S3. Diabetes clinic-related domain outcome ratings from Delphi Surveys 1 and 2. Table S4. Medical domain outcome ratings from Delphi Surveys 1 and 2. Table S5. Blood glucose domain outcome ratings from Delphi Surveys 1 and 2. Table S6. Treatment adherence domain outcome ratings from Delphi Surveys 1 and 2. Table S7: Intervention-related domain outcome ratings from Delphi Surveys 1 and 2. Table S8: Level of support in the final voting phase of the consensus meeting for supplementary important outcomes*. (DOCX 28 kb) [file 13063_2017_2364_MOESM2_ESM.docx]

**Supplementary material**

Title: A Core Outcomes Set for clinical trials of interventions for young adults with type 1 diabetes: an international, multi-perspective Delphi consensus study

Authors:

Molly Byrne^a^, Anthony O’Connell^a^, Aoife M. Egan^b^, Sean F. Dinneen^b,c^, Lisa Hynes^a^, Mary Clare O’ Hara^b,c^, Richard IG Holt^d^, Ingrid Willaing^e^, Michael Vallis^f^, Christel Hendrieckx^g,h^, Imelda Coyne^i^.

Supplementary Table 1: Lifestyle domain outcome ratings* from Delphi survey 1 and 2.

| **Lifestyle outcomes** | **Round 1** (N=132) | |  | **Round 2 (**N = 81) | |  |
| --- | --- | --- | --- | --- | --- | --- |
|  | Mean | SD | | Mean | SD | |
| Body Mass Index (BMI) | 6.07 | 1.99 | | 5.80 | 1.88 | |
| Level of daily physical activity | 7.40 | 1.50 | | 7.49 | 1.28 | |
| Amount of sedentary behaviour (sitting down) | 6.66 | 1.82 | | 6.68 | 1.56 | |
| Detail on food eaten by participants | 7.22 | 1.56 | | 7.04 | 1.36 | |
| Details about meal pattern of participants | 6.86 | 1.96 | | 6.74 | 1.49 | |
| Levels of smoking | 7.09 | 1.98 | | 7.03 | 1.70 | |
| Levels of alcohol consumption | 7.15 | 1.85 | | 7.10 | 1.56 | |
| Levels of recreational drug abuse | 6.79 | 2.09 | | 6.74 | 1.83 | |

* Participants were asked to rate how important they considered each of the outcomes, on a scale of 1-9, where 9 was the most important.

Supplementary Table 2: Quality of life domain outcome ratings from Delphi survey rounds 1 and 2.

| **Quality of life outcomes** | **Round 1** (N=132) | | | |  | **Round 2 (**N = 81) | | | |  |  |
| --- | --- | --- | --- | --- | --- | --- | --- | --- | --- | --- | --- |
|  | Mean | | SD | | | Mean | | SD | | |  |
| Measures of Diabetes related burden or distress | | 8.41 | | 1.18 | | | 8.49 | | 0.85 | | |
| Anxiety Levels | | 7.96 | | 1.36 | | | 7.96 | | 1.13 | | |
| Measures of fears related to diabetes | | 8.24 | | 1.17 | | | 8.18 | | 0.98 | | |
| Depression Levels | | 8.12 | | 1.24 | | | 8.20 | | 1.08 | | |
| Whether a person discussed diabetes with their family more | | 7.16 | | 1.54 | | | 7.23 | | 1.38 | | |
| Measures of quality of life | | 7.99 | | 1.42 | | | 8.10 | | 1.03 | | |
| Perceived social support | | 7.73 | | 1.45 | | | 7.93 | | 1.06 | | |
| Perceived familial support | | 7.76 | | 1.47 | | | 7.87 | | 1.24 | | |
| Perceived support from diabetes care team | | 7.98 | | 1.29 | | | 8.05 | | 0.92 | | |
| Level of comfort in discussing diabetes with their doctor | | 7.81 | | 1.61 | | | 7.71 | | 1.29 | | |
| Level of comfort in seeking medical advice regarding diabetes | | 7.91 | | 1.42 | | | 7.99 | | 1.11 | | |
| Perceived stigma experienced in relation to diabetes | | 7.75 | | 1.51 | | | 7.88 | | 1.13 | | |

Supplementary Table 3: Diabetes clinic-related domain outcome ratings from Delphi survey rounds 1 and 2.

| **Diabetes clinic outcomes** | **Round 1** (N=132) | |  | **Round 2 (**N = 81) | |  |
| --- | --- | --- | --- | --- | --- | --- |
|  | Mean | SD | | Mean | SD | |
| Clinic attendance frequency | 7.08 | 1.65 | | 7.07 | 1.43 | |
| Number of missed clinic appointments | 7.05 | 1.73 | | 6.99 | 1.26 | |
| Percentage of clinic appointments attended | 6.92 | 1.73 | | 6.84 | 1.46 | |
| Number of rescheduled clinic appointments | 5.98 | 1.96 | | 5.80 | 1.52 | |
| Number of cancelled clinic appointments | 6.14 | 1.98 | | 5.98 | 1.37 | |
| Number of contacts other than scheduled clinic appointments | 7.01 | 1.66 | | 7.07 | 1.39 | |
| Number of diabetes self-management education sessions attended | 7.22 | 1.84 | | 7.05 | 1.67 | |
| Number of diabetes self-management education sessions missed | 6.57 | 2.10 | | 6.51 | 1.75 | |
| Attended eye exam as required | 7.73 | 1.39 | | 7.47 | 1.37 | |
| Attended foot exam as required | 7.48 | 1.68 | | 7.31 | 1.38 | |

Supplementary Table 4: Medical domain outcome ratings from Delphi survey rounds 1 and 2.

| **Medical outcomes** | **Round 1** (N=132) | | | |  | **Round 2 (**N = 81) | | | |  |  |
| --- | --- | --- | --- | --- | --- | --- | --- | --- | --- | --- | --- |
|  | Mean | | SD | | | Mean | | SD | | |  |
| Number of instances of diabetic ketoacidosis (DKA) | | 8.45 | | 1.00 | | | 8.48 | | .88 | | |
| Length of DKA | | 7.16 | | 1.87 | | | 7.05 | | 1.51 | | |
| Number of hospital admissions and readmissions for DKA | | 8.30 | | 1.17 | | | 8.35 | | .96 | | |
| Total number of hospitalisations | | 7.63 | | 1.67 | | | 7.54 | | 1.24 | | |
| HbA1c (glycated haemoglobin) measured by a clinician/researcher | | 8.13 | | 1.11 | | | 7.99 | | 1.22 | | |
| Self-reported HbA1c | | 6.37 | | 2.16 | | | 6.23 | | 1.75 | | |
| Number of symptoms related to other aspects of Type 1 Diabetes | | 7.74 | | 1.40 | | | 7.50 | | 1.09 | | |
| Number of deaths | | 7.94 | | 1.74 | | | 8.00 | | 1.09 | | |
| Amount of diabetes related complications | | 8.27 | | 1.11 | | | 8.30 | | .91 | | |
| Levels of urine protein | | 7.50 | | 1.56 | | | 7.49 | | 1.26 | | |
| Blood Pressure | | 7.41 | | 1.67 | | | 7.30 | | 1.44 | | |

Supplementary Table 5: Blood glucose domain outcome ratings from Delphi survey rounds 1 and 2.

| **Blood glucose outcomes** | **Round 1(**N=132) | | | |  | **Round 2 (**N = 81) | | | |  |  |
| --- | --- | --- | --- | --- | --- | --- | --- | --- | --- | --- | --- |
|  | Mean | | SD | | | Mean | | SD | | |  |
| Number of events of hypoglycaemia (low blood sugar) | | 7.95 | | 1.35 | | | 7.80 | | 1.25 | | |
| Severity of events of hypoglycaemia | | 8.38 | | 0.98 | | | 8.35 | | 0.94 | | |
| Number of events of hyperglycaemia (high blood sugar) | | 7.32 | | 1.48 | | | 7.21 | | 1.26 | | |
| Severity of events of hyperglycaemia | | 7.80 | | 1.27 | | | 7.80 | | 1.07 | | |
| Number of symptoms related to hypoglycaemia | | 7.60 | | 1.47 | | | 7.60 | | 1.21 | | |
| Number of symptoms related to hyperglycaemia | | 7.09 | | 1.52 | | | 7.10 | | 1.17 | | |
| Number of hospitalisations for hypoglycaemia | | 8.34 | | 1.06 | | | 8.34 | | .90 | | |
| Number of hospitalisations for hyperglycaemia | | 8.12 | | 1.25 | | | 8.20 | | .89 | | |
| Amount of time spent in normoglycaemia (normal blood sugar) | | 7.60 | | 1.38 | | | 7.33 | | 1.34 | | |
| Amount of time spent in hyperglycaemia | | 7.60 | | 1.36 | | | 7.36 | | 1.30 | | |
| Amount of time spent in hypoglycaemia | | 7.90 | | 1.44 | | | 7.64 | | 1.25 | | |
| Self-reported body cues for hypoglycaemia | | 7.78 | | 1.27 | | | 7.68 | | 1.24 | | |
| Self-reported body cues for normoglycaemia | | 6.70 | | 2.06 | | | 6.39 | | 1.76 | | |
| Self-reported body cues for hyperglycaemia | | 7.09 | | 1.76 | | | 6.98 | | 1.51 | | |

Supplementary Table 6: Treatment adherence domain outcome ratings from Delphi survey rounds 1 and 2.

| **Treatment adherence outcomes** | **Round 1** (N=132) | |  | **Round 2 (**N = 81) | |  |
| --- | --- | --- | --- | --- | --- | --- |
|  | Mean | SD | | Mean | SD | |
| Frequency of blood glucose readings | 7.66 | 1.37 | | 7.68 | 1.13 | |
| Differences between self-reported logs of blood glucose and objective measures of blood glucose | 6.78 | 1.93 | | 6.54 | 1.68 | |
| Adherence to prescribed insulin regimen | 7.57 | 1.61 | | 7.43 | 1.56 | |
| Mean total insulin dosage in 24 hours | 6.18 | 2.13 | | 5.96 | 1.79 | |
| Self-reported insulin administration at different times of the day | 6.81 | 1.90 | | 6.40 | 1.64 | |
| Level of adjusting quick-acting insulin according to carbohydrate content | 7.91 | 1.30 | | 7.59 | 1.27 | |
| Level of using trends in blood glucose readings to adjust long-acting glucose | 7.73 | 1.39 | | 7.61 | 1.14 | |
| Taking correction doses of insulin | 8.00 | 1.13 | | 7.85 | 1.03 | |
| Levels of adjusting insulin-carbohydrate meal/snack ratios | 7.87 | 1.21 | | 7.79 | 1.14 | |
| Adjusting carbohydrate intake or insulin in response to alcohol | 7.73 | 1.29 | | 7.77 | 1.10 | |
| Self-monitoring blood glucose in response to daily life events (eg. Driving, stressful events) | 8.07 | 1.09 | | 8.07 | 1.03 | |
| Testing blood glucose after event of hypoglycaemia | 7.84 | 1.49 | | 7.66 | 1.34 | |
| Testing blood glucose after event of hyperglycaemia | 7.44 | 1.56 | | 7.34 | 1.48 | |
| Whether participants carry a fast and long lasting carbohydrate snack | 7.39 | 1.75 | | 6.44 | 1.50 | |
| Level of checking blood glucose during the night | 6.55 | 1.71 | | 6.44 | 1.50 | |
| Level of rotation of injection sites | 7.38 | 1.66 | | 7.41 | 1.32 | |
| Level of changing of needles | 7.13 | 1.90 | | 6.91 | 1.72 | |
| Level of checking of medication/injection supplies | 6.91 | 1.90 | | 6.83 | 1.56 | |

Supplementary Table 7: Intervention-related domain outcome ratings from Delphi survey rounds 1 and 2.

| **Intervention-related outcomes** | **Round 1** (N=132) | | | |  | **Round 2 (**N = 81) | | | |  |  |
| --- | --- | --- | --- | --- | --- | --- | --- | --- | --- | --- | --- |
|  | Mean | | SD | | | Mean | | SD | | |  |
| Perceived level of barriers to treatment | | 7.78 | | 1.45 | | | 7.73 | | 1.28 | | |
| Perceived benefits of treatment | | 7.93 | | 1.36 | | | 7.97 | | 1.15 | | |
| Perceived costs of treatment | | 6.28 | | 2.21 | | | 6.19 | | 1.97 | | |
| Perceived level of control over diabetes | | 7.95 | | 1.32 | | | 7.84 | | 1.16 | | |
| Knowledge about diabetes | | 7.80 | | 1.44 | | | 7.82 | | 1.12 | | |
| Knowledge about blood glucose | | 7.80 | | 1.38 | | | 7.76 | | 1.07 | | |
| Whether participant incorporates skills learned during the intervention into their daily life | | 8.08 | | 1.17 | | | 8.10 | | 1.06 | | |
| Maintaining skills learned during intervention | | 8.06 | | 1.17 | | | 7.91 | | 1.22 | | |
| Participant feedback on taking part in the study | | 7.39 | | 1.61 | | | 7.30 | | 1.29 | | |
| Participant feedback on intervention | | 7.78 | | 1.41 | | | 7.61 | | 1.23 | | |
| Overall satisfaction with intervention | | 7.80 | | 1.30 | | | 7.67 | | 1.22 | | |
| Adverse events occurring during the study | | 7.80 | | 1.42 | | | 7.75 | | 1.36 | | |
| Any technical issues | | 7.14 | | 1.59 | | | 7.04 | | 1.32 | | |
| Preference of treatment (the intervention or existing treatment) | | 7.72 | | 1.46 | | | 7.56 | | 1.35 | | |

Supplementary Table 8: Level of support in the final voting phase of the consensus meeting for supplementary important outcomes*.

| Outcome | N voting for inclusion of outcome in the final COS (N=12) |
| --- | --- |
| 1. Perceived Social Support | 8 |
| 1. Incorporate skills learned during the intervention into daily life | 7 |
| 1. Perceived support from diabetes care team | 7 |
| 1. Knowledge about diabetes | 6 |
| 1. Maintaining skills learned during intervention | 6 |
| 1. Body mass index (BMI) | 5 |
| 1. Perceived stigma in relation to diabetes | 4 |
| 1. Number of hospitalisations for hypoglycaemia | 3 |
| 1. No. of hospitalisations and readmissions for DKA | 3 |
| 1. Perceived Level of Barriers to Treatment | 3 |
| 1. Levels of adjusting insulin-carbohydrate meal-snack ratios | 3 |
| 1. Anxiety Levels | 3 |
| 1. Amount of Diabetes related complications | 2 |
| 1. No. of deaths | 2 |
| 1. Self-monitoring of blood glucose in response to daily life events | 2 |
| 1. Knowledge about blood glucose | 1 |
| 1. Measure of fear related to diabetes | 1 |
| 1. Perceived benefits of treatment | 1 |
| 1. Depression levels | 0 |
| 1. Quality of life measure | 0 |
| 1. Adverse Events Occurring during study | 0 |
| 1. Number of hospitalisations for hyperglycaemia | 0 |

* ‘Supplementary important outcomes’ were category A outcomes (original and new) which were voted upon during the consensus meeting, but did not make it to the final COS.
